# Supplementary material for: Association between oral microbiome and seven types of cancers in East Asian population: a two-sample Mendelian randomization analysis
Source: Front Mol Biosci. 2023 Nov 21;10:1327893. doi: 10.3389/fmolb.2023.1327893 (PMC10702768; doi:10.3389/fmolb.2023.1327893)
Supplement: Supplementary file 1 [file DataSheet1.ZIP › Supplementary Materials/MR plots of saliva/saliva═╝/Lung Cancer/pheno.2919_to_lung cancer_forest.pdf]

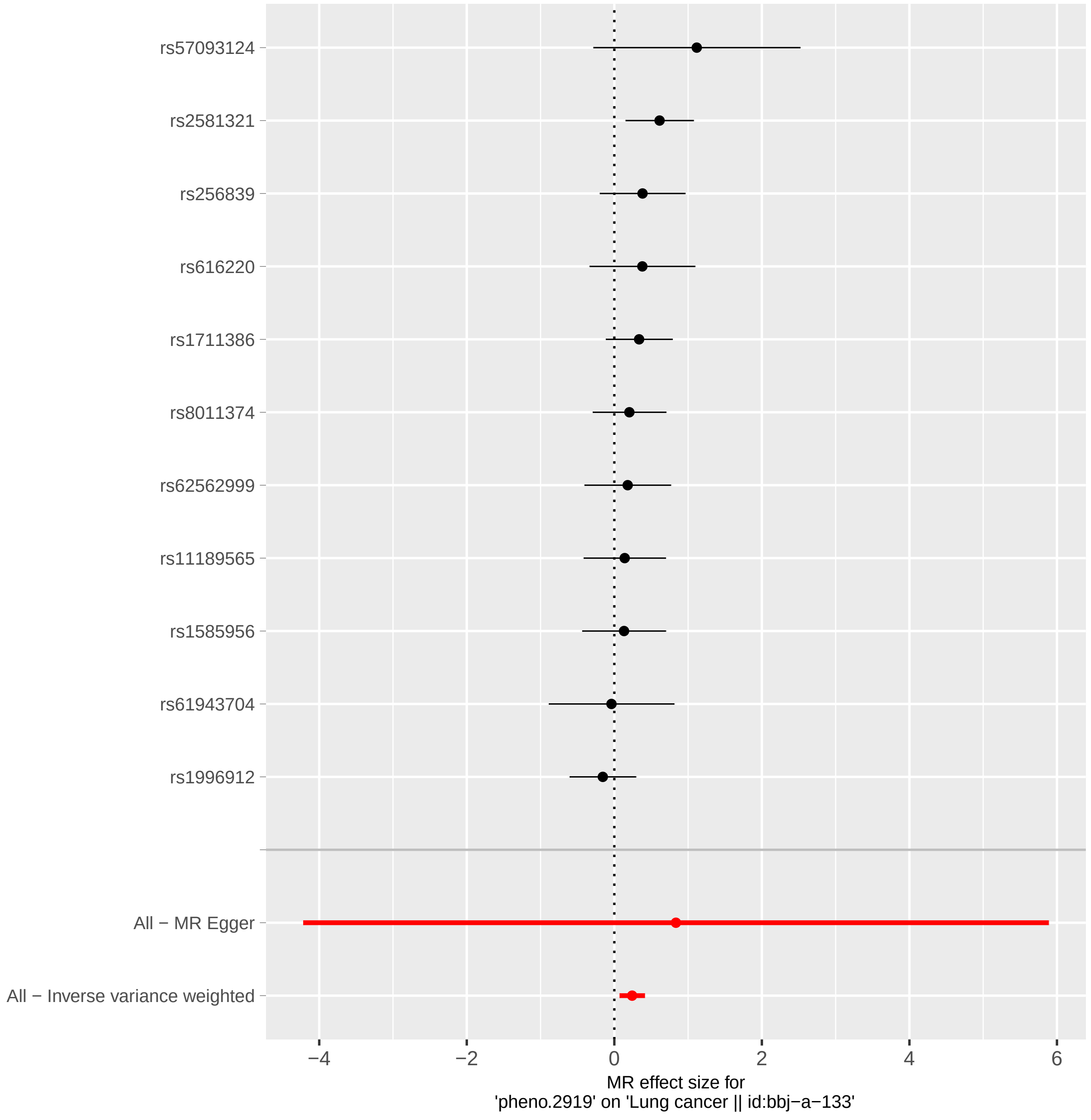
rs57093124
rs2581321
rs256839
rs616220
rs1711386
rs8011374
rs62562999
rs11189565
rs1585956
rs61943704
rs1996912
All − MR Egger
All − Inverse variance weighted
−4
−2
0
2
4
6
MR effect size for
'pheno.2919' on 'Lung cancer || id:bbj−a−133'
